# Tapioca Cardiomyopathy: Curse of Cassava Endomyocardial Fibrosis

**DOI:** 10.14740/cr394w

**Published:** 2015-04-06

**Authors:** Prem Krishna Anandan, Patel Jigarkumar Shukkarbhai, Jimmy George, Prabhavathi Bhatt, Cholenahally Nanjappa Manjunath

**Affiliations:** a Sri Jayadeva Institute of Cardiovascular Science & Research, Bengaluru, Karnataka, India

**Keywords:** Endomyocardial fibrosis, Tapioca, Restrictive cardiomyopathy

## Abstract

Tropical endomyocardial fibrosis is a rare entity in the present era. Restrictive cardiomyopathy due to tapioca consumption is very rare, although it has been reported in India, especially in state of Kerala. We report a rare case of restrictive cardiomyopathy secondary to tapioca consumption in a 20-year-old male patient.

## Introduction

Endomyocardial fibrosis (EMF), though a vanishing entity, is still rampant in tropical developing countries. A number of etiological factors play a role, among which diet deficient in protein, like tapioca (cassava), is one among them.

## Case Report

**Figure 1:**
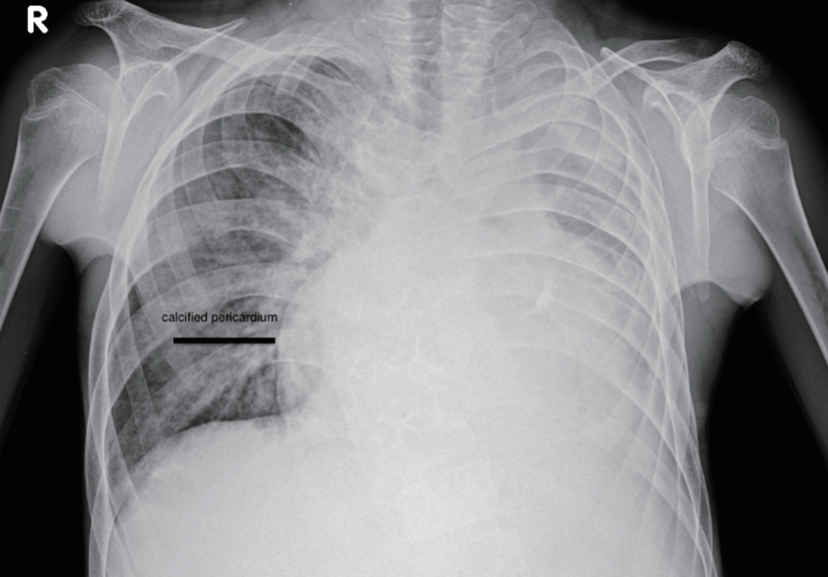
Chest X-ray PA view showing pericardial calcification.

**Figure 2:**
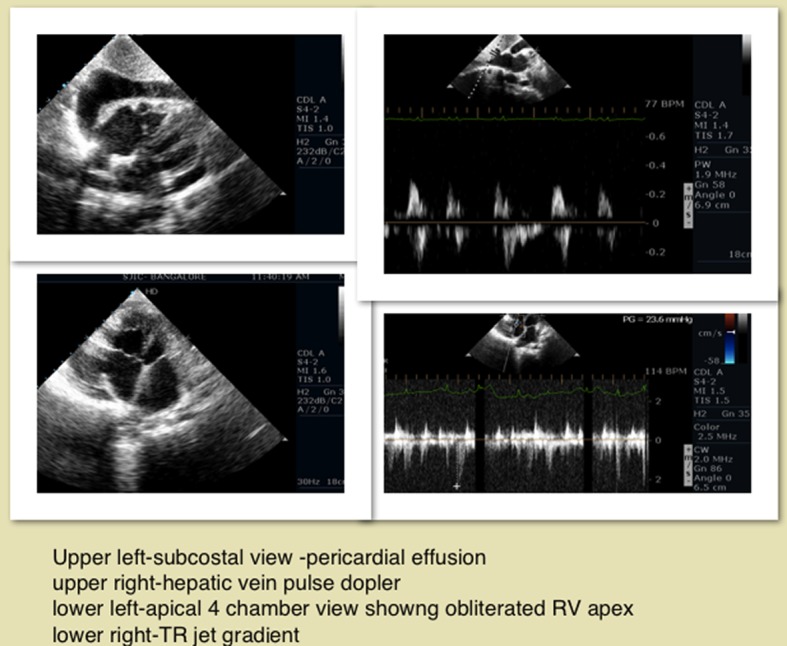
Two-dimensional echocardiogram apical four-chamber, subcostal views showing obliterated RV apex, pericardial effusion, hepatic vein respiratory variation, TR jet.

We report a 20-year-old patient who presented to our institute with abdominal distension, pedal edema and dyspnea NYHA III. On examination, his jugular venous pressure (JVP) was elevated with tender hepatomegaly and ascites. Pulse rate was 90/min irregular, and blood pressure was 100/70 mm Hg. Cardiac examination revealed muffled heart sounds. Complete hemogram was normal with normal eosinophil count, and stool for ova cysts was negative. There was no history of any radiation exposure. ECG showed atrial fibrillation. Chest X-ray showed cardiomegaly with pericardial calcification (Fig. 1). Two-dimensional echocardiogram showed pericardial effusion, biatrial enlargement, and small ventricular cavities with right ventricular (RV) apex hypertrophy (Fig. 2), video 1 showed the apical four-chamber view showing biatrial enlargement, obliterated RV apex; video 2 showed the subcostal four-chamber view showing pericardial effusion (supplementary videos 1, 2, www.cardiologyres.org). There was ventricular interdependence, mild tricuspid regurgitation, and pulmonary artery systolic pressure of 30 mm Hg. Respiratory variation of pulse wave velocities across tricuspid valve was present. All features were suggestive of restrictive cardiomyopathy with RV failure. The patient was treated with diuretics, digoxin and warfarin.

## Discussion

Restrictive cardiomyopathy due to tropical EMF was first reported in Africa. The disease affected children and young adults, with a male preponderance in an epidemic fashion with a geographical distribution within 15° on either side of the equator. Studies identified Kerala as the hot spot for the disease with isolated reports [1]. The clinical and autopsy studies have shown left and right ventricular apical fibrosis, with varying degrees of atrioventricular valve regurgitation. JNP Davies first coined the term EMF. The triad of elevated JVP, ascites and hepatomegaly formed the hallmark of RV EMF, which can present with cyanosis and clubbing because of stretch opening of foramen ovale [2]. Mild cardiomegaly, loud left ventricular third heart sound, short systolic murmur and severe pulmonary hypertension formed the hallmark for the diagnosis of left ventricular EMF. Chest X-rays show varying degrees of cardiomegaly and at times typical endocardial calcification. RV EMF with severe right atrial dilatation is reflected in the electrocardiogram as qR pattern in V1 [3]. Typical Doppler and two-dimensional features are biatrial dilatation, spontaneous intracavitary echos, and formation of thrombi [4]. Sub-endocardial fibrosis affects the apices and the inflow tract of the right or left ventricle, or both [5]. At gross examination, the ventricles look small with grossly dilated atria, and thrombi are usually seen in the atria denoting stagnation and atrial fibrillation. The pericardium looks normal but pericardial effusion and ascites are common findings. The fibrotic retraction of the RV apex produces the typical apical dimple [6]. The early part of the disease is rarely clinically recognized in India and the disease comes to attention in the late stages and isolated endocardial involvement and intracardiac thrombi are the peculiar features. Davies has described three phases of the disease [7]. The initial phase is an acute carditis phase, characterized by febrile illness and in severe cases with heart failure and shock followed by a progress into a subacute phase followed by a chronic phase. Most of the patients come to clinical attention in this chronic burnt-out phase. Suggested etiologies include association with malaria, toxoplasma infection, rheumatic fever, chronic beriberi, loa loa infection, Loffler’s endocardial disease and lymphatic obstruction due to filariasis. The cyanogenic glycosides in tapioca, and *Argemone mexicana*, high vitamin D and cerium content of tapioca, serotonin in plantain were the toxic agents evaluated and found to cause EMF [8]. Induced protein deficiency with dietary excess of carbohydrates provided by banana and tapioca rich diets was used to induce animal models [9]. In our patient we postulated the cause as tapioca since all other hematological investigations were normal. The patient came from a very low socioeconomic status with tapioca being the major portion of his diet. His father also expired due to some cardiac ailment, with similar features, the cause of which was not evaluated. Irrespective of intense multifaceted research, EMF continues to be an enigmatic disorder. The specific endocardial involvements, localization to certain geographical pockets, propensity to affect the poor and typical endocardial calcification are the peculiarities of this disease.

### Conclusion

To conclude EMF remains one of the most neglected diseases worldwide, and research into its pathophysiological mechanisms will probably improve outcomes and alter the natural history of the disease. Tapioca-induced EMF should be kept in mind when we come across cases from endemic areas.

### Learning objective

In tropical countries still cassava diet should be kept in mind as an etiology of restrictive cardiomyopathy since it is considered a “poor man’s food”.
